# Deciphering the Differences Between Epstein–Barr Virus‐Associated and Negative Gastric Cancer in the Prospect of *CDKN2A* Genomic Alterations and Lymphoid Infiltration

**DOI:** 10.1002/cam4.70409

**Published:** 2025-01-22

**Authors:** Fuda Xie, Bonan Chen, Yang Lyu, Peiyao Yu, Canbin Fang, Kam Tong Leung, Shouyu Wang, Dazhi Xu, Jun Yu, Kwok Wai Lo, Ka Fai To, Wei Kang

**Affiliations:** ^1^ Department of Anatomical and Cellular Pathology, State Key Laboratory of Translational Oncology, Sir Y.K. Pao Cancer Center, Prince of Wales Hospital The Chinese University of Hong Kong Hong Kong SAR China; ^2^ Institute of Digestive Disease, State Key Laboratory of Digestive Disease, Li Ka Shing Institute of Health Science The Chinese University of Hong Kong Hong Kong SAR China; ^3^ CUHK‐Shenzhen Research Institute Shenzhen China; ^4^ Department of Pediatrics The Chinese University of Hong Kong Hong Kong SAR China; ^5^ Department of Hepatobiliary Surgery The Affiliated Drum Tower Hospital of Nanjing University Medical School Nanjing China; ^6^ Department of Gastric Surgery, Fudan University Shanghai Cancer Center; Department of Oncology, Shanghai Medical College Fudan University Shanghai China; ^7^ Department of Medicine and Therapeutics The Chinese University of Hong Kong Hong Kong SAR China

**Keywords:** CD8^+^ T cell infiltration, CDKN2A, Epstein–Barr virus, gastric cancer

## Abstract

**Background:**

Gastric cancer (GC) is a major health concern worldwide. One important contributing factor is the presence of the Epstein‐Barr virus (EBV). However, the molecular pattern of how EBV participates in the malignant transition process remains unclear.

**Methods:**

GC samples were stained by immunohistochemistry, fluorescent and EBV‐encoded small RNA in situ hybridization to identify CD8 expression, CDKN2A genomic alteration, and EBV existence. Functional potentials of EBV infection were predicted by bioinformatic enrichment analysis.

**Results:**

CDKN2A genestayed intact in all EBV‐associated GC cases. Meanwhile, CDKN2A deletion (8.43% cases) was exclusive to EBV‐negative GC cases. Furthermore, EBV infection was positively correlated with CD8+T cell infiltration, and both of them predicted better prognosis.

**Conclusion:**

This study highlighted the comprehensive impact of EBV infection in GC formation and proposed a thought‐provoking observation for further investigation into the roles of CDKN2A and EBV infection in gastric tumorigenesis.

**Abbreviations**
BBC3: BCL2 binding component 3
BCL2L11: Bcl‐2‐like protein 11
CDKN2A: Cyclin‐Dependent Kinase Inhibitor 2A
CDKN2B: Cyclin‐Dependent Kinase Inhibitor 2B
CIN: chromosomal instability
CNV: copy number variations
DEG: differentially expressed gene
EBER: EBV‐encoded small RNA
EBV: Epstein–Barr virus
EBVaGC: EBV‐associated gastric cancer
EBVnGC: EBV‐negative gastric cancer
FFPE: formalin‐fixed paraffin‐embedded
FISH: fluorescent in situ hybridization
GC: gastric cancer
GO: Gene Ontology
GS: genomically stable
GSEA: gene set enrichment analysis
HOXA10: Homeobox A10
IHC: immunohistochemistry
KEGG: Kyoto Encyclopedia of Genes and Genomes
MDM2: E3 Ubiquitin‐Protein Ligase Mdm2
MLH1: MutL Homolog 1
MSI: microsatellite instability
NPC: nasopharyngeal cancer
POLE: POLE mutation
STAD: stomach adenocarcinoma
TCGA: The Cancer Genome Atlas

## 1 Introduction

Gastric cancer (GC) arises as a major cancer burden worldwide, especially in Eastern Asia. There were over one million new cases in 2020 which resulted in 768,793 deaths, and 435,211 were from Eastern Asia [1]. While numerous factors contribute to the development of GC, one infectious agent that has garnered attention is the Epstein–Barr virus (EBV). As a member of the human herpesvirus family, EBV plays a pivotal role in approximately 5%–10% of GC cases [2]. The virus has been implicated in promoting the transformation of epithelial cells into malignant cancer cells [3, 4], as well as assisting gastric cancer cells in evading immune surveillance [5]. Given the significance of EBV inherence and the lack of mechanistic understanding of the oncogenic pattern of EBV, it is vital to retrieving a better understanding of this carcinogenic factor.

Inspired by the former studies demonstrating that the low cyclin‐dependent kinase inhibitor 2A (CDKN2A) expression in EBV‐associated gastric tumorigenesis [6], we recently explored the potential connection between the genomic and expression alteration of *CDKN2A* and the EBV infection status in gastric cancer and obtained several thought‐provoking results. *CDKN2A* is recognized to be a vital tumor suppressor gene that is frequently deleted or mutated in multiple tumorigeneses [7, 8, 9]. The gene is located at position 21 on human chromosome 9, consisting of three exons and two introns, and encodes tumor suppressor proteins named p16 and p14ARF. p16 plays a role in inhibiting cyclin‐dependent kinases CDK4 and CDK6 and regulates the cell cycle by slowing down the progression from the G1 phase to the S phase [10]. On the other hand, the loss of p14ARF due to a mutation in *CDKN2A* can lead to an elevated level of E3 ubiquitin–protein ligase Mdm2 (MDM2), ultimately resulting in the loss of p53 function and control over the cell cycle [11]. In this work, we demonstrated the association between genomic stability and epigenetic modification of *CDKN2A* and EBV infection by both public database and in‐house cohort. Furthermore, we discussed the relationships between EBV infection and CD8^+^ T cell infiltration and their potential for predicting GC patient prognosis. Lastly, we proposed an interesting observation regarding the impact of *CDKN2A* genomic alteration or methylation level on immune response for further investigation.

## 2 Methods and Materials

### 2.1 Human Tissue Samples

A GC tissue microarray (Hong Kong cohort) was used in this study, containing 264 cases of GC tumor collected from Prince of Wales Hospital. The study was approved by the Joint Chinese University of Hong Kong‐New Territories East Cluster Clinical Research Ethics Committee (CREC Ref. No. 2021‐083). A waiver of the consent form was granted by the Ethics Committee.

### 2.2 Immunohistochemistry (IHC) and EBV‐Encoded Small RNA (EBER) In Situ Hybridization

The Ventana BenchMark ULTRA auto‐stainer (Ventana Corporation) was used to conduct immunohistochemistry staining on tissue microarrays. Each sample had a 4‐μm‐thick section taken from a formalin‐fixed paraffin‐embedded (FFPE) tissue specimen, which was then deparaffinized, rehydrated, and rinsed in distilled water. Antigen retrieval was done using a pressure cooker with ethylenediaminetetraacetic acid. The sections were incubated with CD8 monoclonal antibody (372902, BioLegend) overnight at 4°C, followed by exposure to horseradish peroxidase (HRP)‐conjugated secondary antibody. The expression level of CD8 was visualized using the Liquid DAB+ Substrate Chromogen System kit (catalog number #K3468, Dako North America, California, USA) and counterstained with hematoxylin (catalog number #G1004, Servicebio, Wuhan, China). The EBV status of the same TMA was evaluated by EBER‐RNA in situ hybridization using RISH Epstein–Barr (EBER) Hybridization Probe (RPI0001T, Biocare Medical). Primary samples with more than 25% EBER‐positive cancer cells were defined as EBVaGC cases, and the rest were described as EBVnGC cases. Similarly, primary samples with more than 25% CD8‐positive cancer cells were defined as CD8‐positive cases, and the rest were described as CD8‐negative cases. The clinicopathologic profiles of each case in the in‐house cohort are recorded in Table S4.

### 2.3 Fluorescent In Situ Hybridization (FISH)

FISH analysis was performed on FFPE tissue sections using a probe specific to the CDKN2A locus (SPEC CDKN2A/CEN 9 Dual Color Probe [PL22], ZytoLight). Initially, 4‐μm‐thick sections that were obtained from FFPE tissue specimens were deparaffinized in xylene, dehydrated in graded alcohol, immersed in pretreatment solution, and protease treatment. Subsequently, in situ hybridization was performed using a CDKN2A probe, which hybridized the CDKN2A locus with SpectrumRed. Additionally, a reference probe was introduced, hybridizing the centromere locus with SpectrumGreen. After applying the probe mixture, slides were denatured and incubated at 37°C overnight for hybridization. Upon completion of hybridization, slides underwent washing with washing buffer, followed by counterstaining with 4′,6‐diamidino‐2‐phenylindole.

### 2.4 Gene Expression, Methylation, Functional Enrichment Analysis, and Gene Set Enrichment Analysis (GSEA)

Gene expression and methylation profiling data of CDKN2A in GC tumors and adjacent normal samples were retrieved and then analyzed based on the Cancer Genome Atlas (TCGA) stomach adenocarcinoma (STAD) cohort. The subsequence gene ontology (GO) functional enrichment analysis, Kyoto Encyclopedia of Genes and Genomes (KEGG) pathway analysis, and GSEA were performed by applying R package “ClusterProfiler” [12].

### 2.5 Statistical Analysis

Student's *t*‐test or one‐way ANOVA test was used to analyze statistical differences among assay groups. Statistical results collected in the study were performed by SPSS software version 22.0 (Armonk, NY, USA). Kaplan–Meier survival analysis and log‐rank test were performed to evaluate the effect of EBV infection and CD8 expression on survival. A two‐tailed *p* value less than 0.05 was defined as statistically significant, while a *p* value less than 0.01 was deemed highly significant.

## 3 Results

**FIGURE 1:**
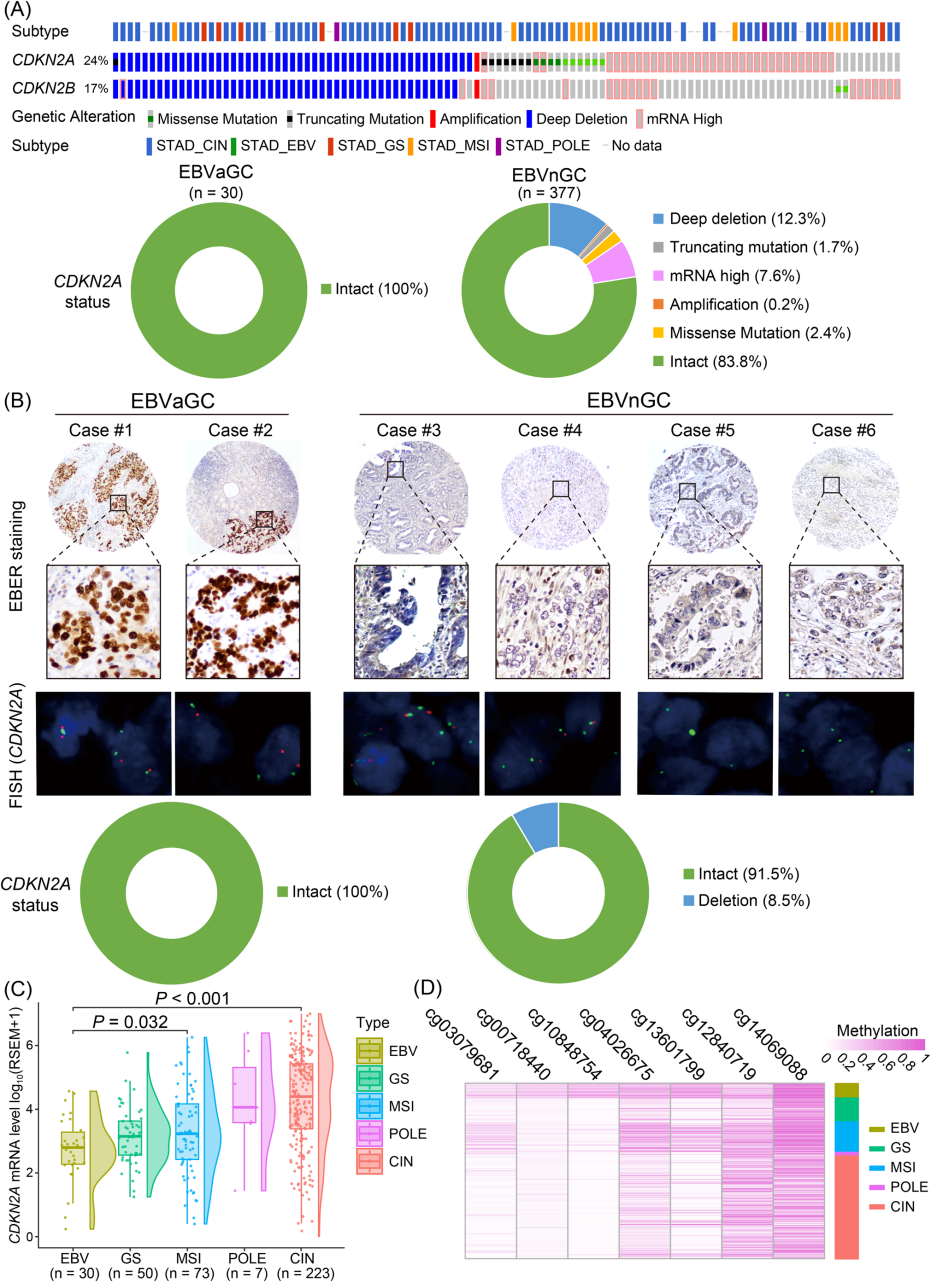
*CDKN2A* homozygous deletion occurs in EBVnGCs, while *CDKN2A* hypermethylation occurs in EBVaGCs. (A) Genetic alterations (gene mutation, deletion, or amplification) and abnormal mRNA expression of *CDKN2A* and *CDKN2B* in subgrouped GC samples from TCGA cohort. (B) Representative images of EBER ISH staining and *CDKN2A* FISH in EBVaGC and EBVnGC samples reveal the fact that *CDKN2A* deletion is only observed in EBVnGC cases. (C) The mRNA levels of *CDKN2A* are significantly downregulated in EBVaGCs in TCGA cohort. (D) The promoter methylation levels of the *CDKN2A* gene are relatively higher in EBVaGCs.

In the beginning, we observed that *CDKN2A* deletion frequently occurred in GC, leading to the dysfunction of *CDKN2A* and the malignant transformation of GC [13]. As we dug into TCGA cohort, we found that multiple types of genomic alterations, such as homozygous deletion, truncating mutation, missense mutation, and amplification, were harvested in GC samples (Figure 1A). Due to the proximity of chromosomal locations [14], synchronous deletion of cyclin‐dependent kinase inhibitor 2B (CDKN2B) was observed in the same GC samples with *CDKN2A* deletion. However, samples equipped with *CDKN2A* mutation presented no *CDKN2B* mutation, indicating the independency between the mutation pattern of these two tumor suppressor genes. Meanwhile, several samples had high mRNA expression levels of *CDKN2A*, which may result in contrary phenotypes. The samples in TCGA were subgrouped into five subtypes according to their molecular signatures: chromosomal instability (CIN), genomically stable (GS), microsatellite instability (MSI), POLE mutation (POLE), and EBV positive [15]. Interestingly, all four subtypes of GC except EBV positive, as named in EBV‐negative GC (EBVnGC), harvested *CDKN2A* homozygous deletion or mutation. On the contrary, the *CDKN2A* gene stayed intact in EBV‐associated GC samples (EBVaGC). To concrete this novel perspective generated from bioinformatic analysis, we then further investigated the copy number variation (CNV) of *CDKN2A* by EBER in situ hybridization staining and FISH (Figure 1B). As demonstrated in the results, all cases (12/12) with positive EBER staining (EBVaGC) exhibited intact *CDKN2A* genes without any changes in copy number. On the other hand, 91.5% of EBVnGC cases (151/165) had intact *CDKN2A* genes without any changes in copy number. However, 8.48% of the cases (14/165) showed *CDKN2A* gene deletion. These results confirmed that the CNVs of *CDKN2A* much more frequently occur in EBVnGC rather than EBVaGC.

We then further investigated the difference among these five subtypes of GC in the mRNA level of *CDKN2A*. Surprisingly, the expression level of *CDKN2A* was comparably the highest in CIN samples but not in EBV samples. What is even more eye‐catching is that EBVaGC exhibited the lowest *CDKN2A* mRNA level among all GC samples (Figure 1C). So what caused this *CDKN2A* downregulation while it is protected from genomic mutation in EBVaGC? Inspired by the high frequency of aberrant DNA methylation occurrence in EBV‐associated cancers [16, 17], we analyzed the details of *CDKN2A* methylation status in all six CpG islands and revealed its diversity among different kinds of GCs (Figure 1D). As the data show, the methylation level is relatively higher in EBVaGC samples in all methylation sites of *CDKN2A*. More prominently, two sites, cg00718440 and cg10848754, were predominantly methylated in EBVaGC samples.

**FIGURE 2:**
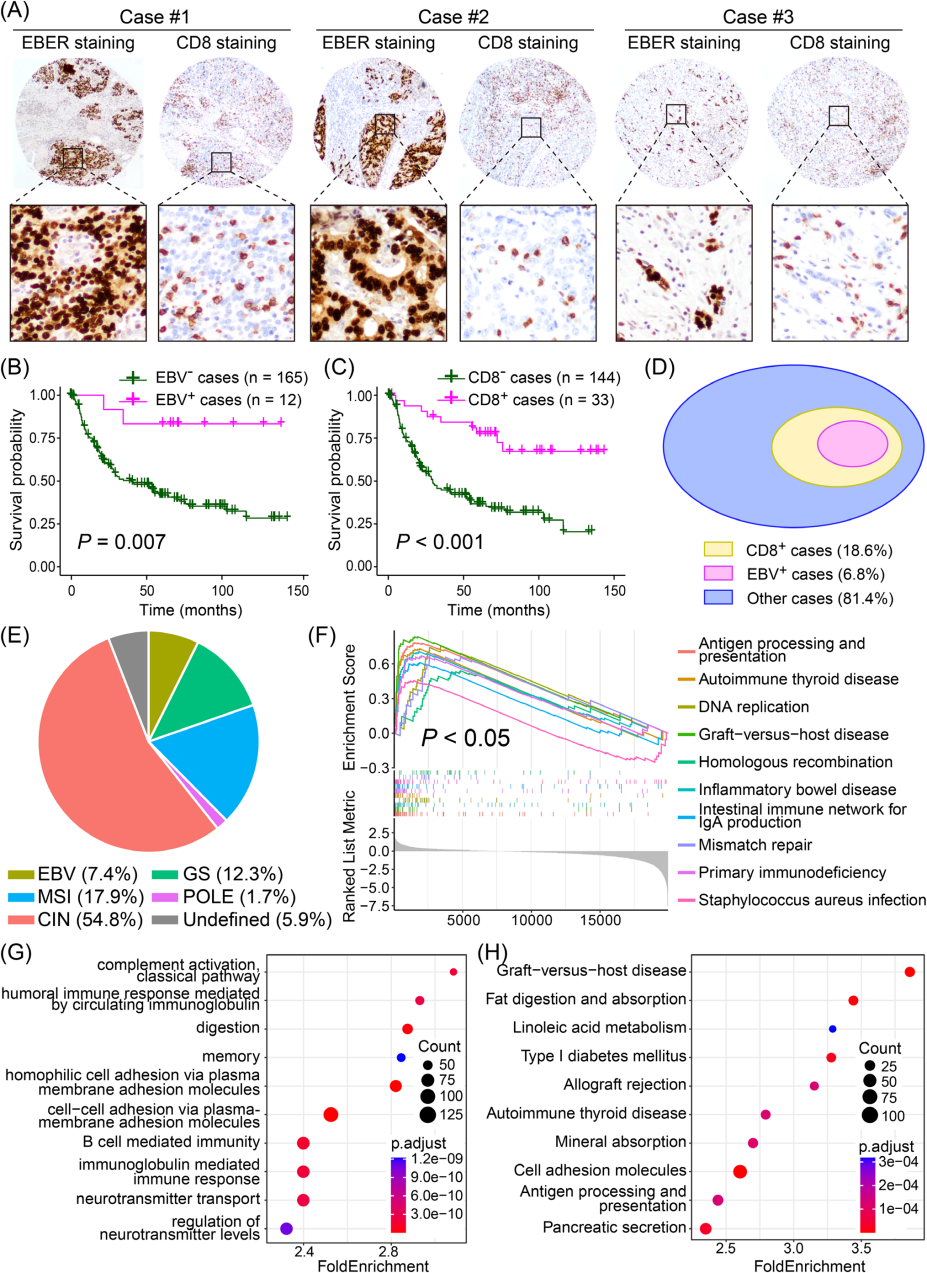
EBVaGC is associated with a higher degree of CD8^+^ T cell infiltration and a better prognosis. (A) Representative images of EBER ISH staining and CD8 IHC staining in EBCaGC samples. (B and C) EBV infection and CD8^+^ T cell infiltration are both associated with better overall survival rates in the Hong Kong cohort. (D) EBV infection presents in all CD8^+^ cases in the Hong Kong cohort, but not all CD8^+^ cases are associated with EBV infection. (E) The proportion of five subtypes of GC in TCGA cohort. (F–H) GSEA, GO enrichment analysis, and KEGG enrichment analysis for EBV‐associated samples compared with other subtypes in TCGA cohort.

After uncovering the impact of EBV infection on the inhibition of *CDKN2A* genomic deletion and the promotion of *CDKN2A* mRNA methylation, we conducted bioinformatic analyses to compare *CDKN2A* genomic intact GC samples with *CDKN2A* deleted cases (Figure S1). The results of biological process enrichment revealed significant influences on host immunity due to the genome stability of *CDKN2A*, evidenced by patterns such as B cell/complement activation, leukocyte‐mediated immunity, and immunoglobulin production. As the EBVaGCs are assumed to have a high degree of lymphoid infiltration [18, 19] and have been recently proven to be correlated with multiple miRNAs contributing to the expression level of PD‐L1 [20, 21], we further investigated the relationship between EBV infection and CD8 expression level. EBER and CD8 IHC staining were performed on serial sections of 177 GC histological specimens in the Hong Kong cohort (Figure 2A). Consistent with the former EBER staining results, 12 samples were identified as EBVaGC, and they demonstrated a higher cumulative survival than the EBVnGCs in the subsequent survival analysis (Figure 2B). Meanwhile, 33 samples were identified as CD8^+^ GC, predicting better overall survival probability (Figure 2C) and shows a protective effect against other hazard factors in Cox multivariate analysis (Figure S2). Interestingly, all 12 EBVaGCs were included in CD8^+^ samples (Figure 2D), providing a new perspective on the correlation between EBV infection and CD8^+^ T cell infiltration in gastric tumorigenesis.

To further describe the transcriptomic landscape of EBVaGC and the alteration against EBVnGC, we constructed GSEA by utilizing TCGA dataset. The differentially expressed genes (DEGs) were generated by comparing the expression level of a certain gene in two groups: EBV (as EBVaGC) and the others (as EBVnGC) (Figure 2E). The top 10 enriched biological processes or signaling pathways ranked by fold enrichment are presented in Figure 2F–H, and the whole result of enrichment analysis is recorded in Tables S1–S3. The GSEA result revealed that EBVaGC samples had a significantly higher expression of genes involved in host immunity, such as antigen processing and presentation, autoimmune thyroid disease, inflammatory bowel disease, and graft‐versus‐host disease. Meanwhile, EBVaGC is positively associated with DNA replication and mismatch repair genes (Figure 2F). GO analysis revealed that EBVaGC had been associated with complement activation classical pathway, humoral immune response, B‐cell‐mediated immunity, and other related processes. It is also associated with other metabolic functions, such as digestion and neurotransmitter transport (Figure 2G). In KEGG analysis, EBVaGC was found to significantly alter various metabolic pathways, such as fat digestion and absorption, linoleic acid metabolism, mineral absorption, type I diabetes mellitus, and so on. Specific immune‐related pathways are also altered, including graft‐versus‐host disease, allograft rejection, and autoimmune thyroid disease (Figure 2H). Generally speaking, DEGs of EBVaGC were predominantly enriched in immune‐related pathways, indicating the activation of immune response when the tumor microenvironment was encountering EBV infection.

## 4 Discussion

The predominant mechanism in EBVaGC is the potential of EBV infection to cause abnormal DNA methylation, resulting in the transcriptional silencing of tumor suppressor genes. Methylation of both host and viral DNA is critical for the development of GC: Host cell DNA methylation may lead to the inactivation of tumor suppressor genes and tumor‐associated antigens, while viral DNA methylation affects the expression of EBV latent and lytic genes [22]. Studies have revealed a high frequency of promoter methylation in the CpG island of host DNA genes, including *CDKN2A*, Homeobox A10 (HOXA10), and MutL Homolog 1 (MLH1) [23]. In addition, EBV was proved to produce microRNAs that repress cellular proteins, such as BCL2 binding component 3 (BBC3) and Bcl‐2‐like protein 11 (BCL2L11), which may ultimately contribute to gastric carcinogenesis through the regulation of apoptosis [2]. Consistent with former findings, our data demonstrated that the methylation level of *CDKN2A* is higher in EBVaGC samples, while the frequently occurring *CDKN2A* deletion was exempted in the same group. For the first time, our study highlights that all EBVaGC cases exhibit intact CDKN2A, a stark contrast to nasopharyngeal cancer (NPC), where over 30% of cases show CDKN2A deletion, particularly in EBV‐positive instances. Although interesting, the detailed mechanism by which the EBV infection “protects” the genomic stability of *CDKN2A* remains unclear and calls for further investigation.

As proved in the FISH results and the promoter methylation level in all six CpG islands of the *CDKN2A* gene in TCGA, EBV infection may protect *CDKN2A* from genomic alterations but also cause promoter hypermethylation of *CDKN2A*. When we combine the fact that EBV infection is associated with both immune response and *CDKN2A* hypermethylation, we can naturally conjecture that *CDKN2A* downregulation would to some extent related to immune activation. However, as we constructed a similar transcriptomic analysis on *CDKN2A* deleted and intact samples, the results revealed that *CDKN2A* deletion positively correlates with immune system dysfunction. As shown in the GSEA diagram, multiple immune‐related biological processes were significantly downregulated in *CDKN2A* homozygous deleted samples. Consistently, recent works also highlighted the driving function of *CDKN2A* in immune infiltration of hepatocellular carcinoma [24] and tumor‐immune microenvironment formation of urothelial carcinoma [25]. To conclude, hypermethylation and homozygous deletion can both result in the downregulation of *CDKN2A* mRNA level. However, the same low *CDKN2A* expression level resulting from different causes could correlate with an utterly contrary phenotype.

On the other hand, the results of the GO enrichment analysis revealed that EBVaGC is associated with various metabolic functions including digestion, neurotransmitter transport, and potassium ion transport. Additionally, the KEGG analysis demonstrated significant alterations in several metabolic pathways in EBVaGC, such as fat digestion and absorption, linoleic acid metabolism, mineral absorption, and type I diabetes mellitus. These findings may be attributed to the inhibition of *CDKN2A* mRNA production by EBV infection, resulting in a decrease in the production of key cell cycle regulators p16 and p14ARF. Previous studies also indicated that the loss of p16 and p14ARF can disrupt normal cell cycle function and lead to alterations in different metabolic pathways within the body [11]. Moreover, the KEGG enrichment analysis conducted on CDKN2A‐deleted samples further supports this assumption, as it revealed changes in metabolic pathways such as the JAK–STAT signaling pathway, cAMP signaling pathway, and calcium signaling pathway. Furthermore, as indicated by the positive association of EBVaGC with DNA replication and mismatch repair genes in the GSEA analysis, the inhibition of cell cycle regulation may have an impact on other oncogenic pathways.

## 5 Conclusions

Overall, our findings demonstrated the upregulated molecular signatures of EBVaGC in *CDKN2A* genomic stability, methylation level, and CD8^+^ T cell infiltration degree. Meanwhile, we observed that the same low *CDKN2A* expression level resulting from different causes, such as hypermethylation and homozygous deletion, could be correlated with contrary phenotypes. This contradictory observation highlights the necessity for further investigation to improve our comprehension of the roles played by *CDKN2A* and EBV infection in various conditions of gastric tumorigenesis.

## Author Contributions

**Fuda Xie:** data curation (equal), formal analysis (equal), software (equal), visualization (equal), writing – original draft (equal). **Bonan Chen:** data curation (equal), investigation (equal), methodology (equal), validation (equal). **Yang Lyu:** data curation (equal), formal analysis (equal), visualization (equal), writing – original draft (equal). **Peiyao Yu:** data curation (equal), formal analysis (equal). **Canbin Fang:** data curation (equal), investigation (equal). **Kam Tong Leung:** conceptualization (equal), methodology (equal), supervision (equal). **Shouyu Wang:** methodology (equal), resources (equal). **Dazhi Xu:** investigation (equal), methodology (equal). **Jun Yu:** methodology (equal), supervision (equal). **Kwok Wai Lo:** methodology (equal), supervision (equal), writing – review and editing (equal). **Ka Fai To:** conceptualization (equal), methodology (equal), supervision (equal), writing – review and editing (equal). **Wei Kang:** conceptualization (equal), funding acquisition (equal), methodology (equal), resources (equal), supervision (equal).

## Ethics Statement

The use of human samples was approved by the Joint Chinese University of Hong Kong‐New Territories East Cluster Clinical Research Ethics Committee, Hong Kong (CREC Ref. No. 2021‐083). A waiver of the consent form was granted by the Ethics Committee.

## Conflicts of Interest

The authors declare no conflicts of interest.

## Supporting information


Figure S1.

Figure S2.

Table S1.
Table S2.
Table S3.

Table S4.

## Data Availability

The datasets used and/or analyzed during the current study are available from the corresponding author on reasonable request.
