# Supplementary material for: Deciphering the Differences Between Epstein–Barr Virus‐Associated and Negative Gastric Cancer in the Prospect of CDKN2A Genomic Alterations and Lymphoid Infiltration
Source: Cancer Med. 2025 Jan 22;14(2):e70409. doi: 10.1002/cam4.70409 (PMC11754542; doi:10.1002/cam4.70409)
Supplement: Supplementary file 1 — Figure S1. [file CAM4-14-e70409-s001.pdf]

Figure S1

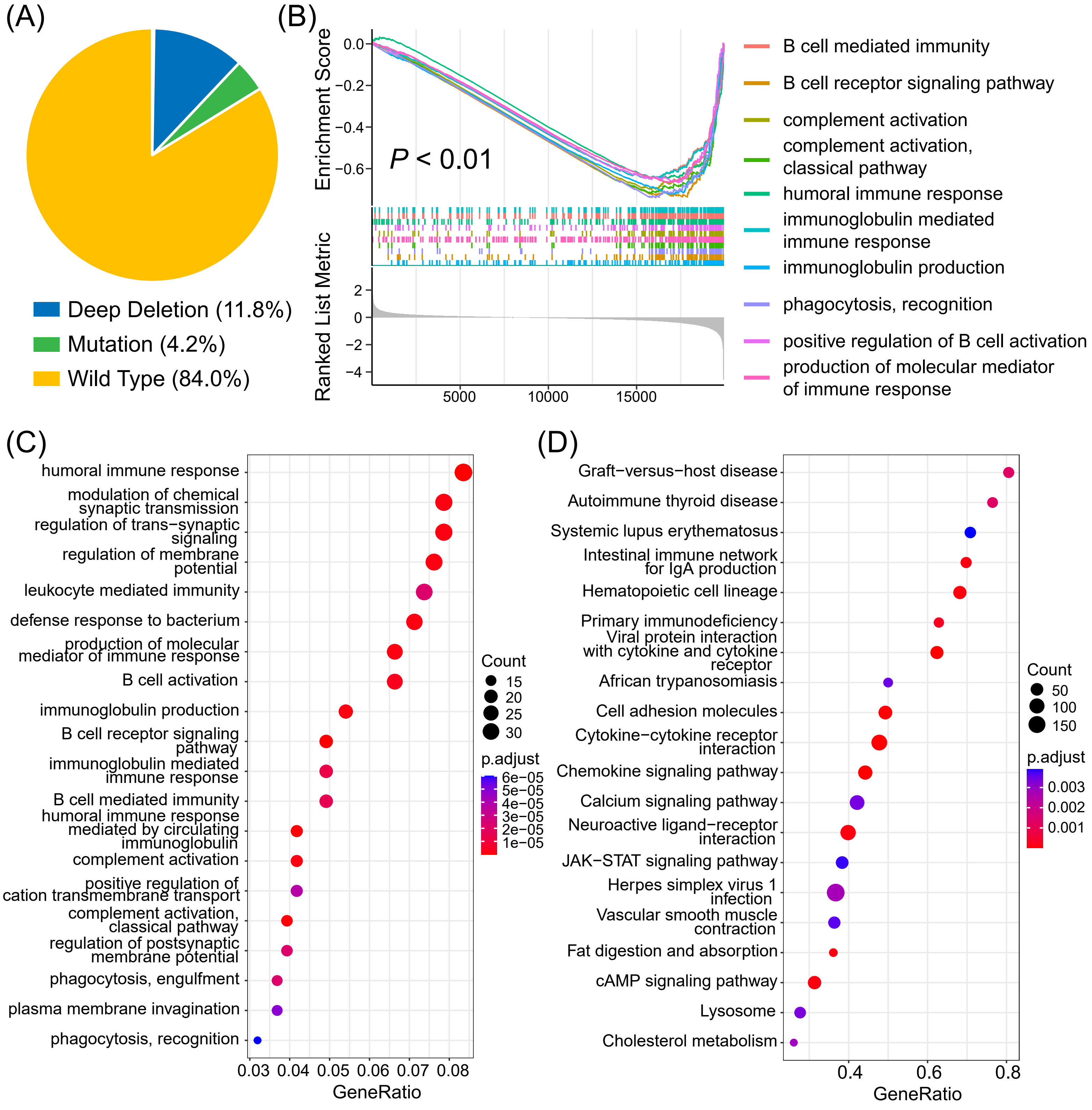

Figure S1: Biological pathways implicated in *CDKN2A*-deleted gastric cancer. (A) Proportion of *CDKN2A*-deleted and -mutated samples in TCGA cohort. (B, C, D) GSEA, GO, and KEGG enrichment analysis for *CDKN2A*-deleted samples compared with wildtype GCs in TCGA.
