# Supplementary figures and images for: Deciphering the Differences Between Epstein–Barr Virus‐Associated and Negative Gastric Cancer in the Prospect of CDKN2A Genomic Alterations and Lymphoid Infiltration
Source: Cancer Med. 2025 Jan 22;14(2):e70409. doi: 10.1002/cam4.70409 (PMC11754542; doi:10.1002/cam4.70409)

Figure S2

## Hazard ratio

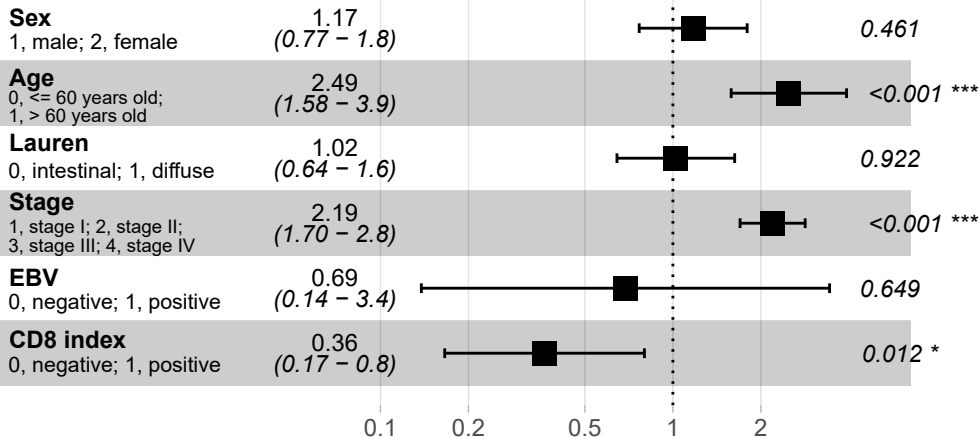

# Events: 93; Global p-value (Log-Rank):  $5.5076e-15$

AIC: 795.12; Concordance Index: 0.76

Supplement: Supplementary file 2 — Figure S2. [file CAM4-14-e70409-s004.pdf]
